# Adoption and use of guidelines for whiplash: an audit of insurer and health professional practice in New South Wales, Australia

**DOI:** 10.1186/s12913-018-3439-5

**Published:** 2018-08-08

**Authors:** Aila Nica Bandong, Andrew Leaver, Martin Mackey, Rodney Ingram, Samantha Shearman, Christen Chan, Ian D Cameron, Niamh Moloney, Rebecca Mitchell, Eoin Doyle, Emma Leyten, Trudy Rebbeck

**Affiliations:** 1 Faculty of Health Sciences, The University of Sydney, 75 East Street, Sydney, Australia; 2 College of Allied Medical Professions, University of the Philippines, Manila, Philippines; 3 John Walsh Centre for Rehabilitation Research, Royal North Shore Hospital, Sydney, Australia; 4 Faculty of Medicine and Health Sciences, Macquarie University, Sydney, Australia; 5 Macquarie University, Australian Institute of Health Innovation, Sydney, Australia; 6 Musculoskeletal Lab/Refshauge Lab (S218), Faculty of Health Sciences, The University of Sydney – Cumberland Campus, 75 East Street, Lidcombe, NSW 2141 Australia

**Keywords:** Guideline adherence, Professional practice, Insurance audits, Whiplash injury, Compliance, Clinical practice guidelines, Primary care

## Abstract

**Background:**

In Australia, the New South Wales (NSW) State Insurance Regulatory Authority has been continuously developing and implementing clinical practice guidelines to address the health and economic burden from whiplash associated disorders (WAD). Despite this, it is uncertain the extent to which the guidelines are followed. This study aimed to determine insurer and health professional compliance with recommendations of the 2014 NSW clinical practice guidelines for the management of acute WAD; and explore factors related to adherence.

**Methods:**

This was an observational study involving an audit of 288 randomly-selected claimant files from 4 insurance providers in NSW, Australia between March and October 2016. Data extracted included demographic, claim and injury details, use of health services, and insurer and health professional practices related to the guidelines. Analyses involved descriptive statistics and correlation analysis.

**Results:**

Median time for general practitioner medical consultation was 4 days post-injury and 25 days for physical treatment (e.g. physiotherapy). Rates of x-ray investigations were low (21.5%) and most patients (90%) were given active treatments in line with the guideline recommendations. The frequency of other practices recommended by the guidelines suggested lower guideline adherence in some areas such as; using the Quebec Task Force classification (19.9%); not using specialised imaging for WAD grades I and II (e.g. MRI, 45.8%); not using routine passive treatments (e.g. manual therapy, 94.0%); and assessing risk of non-recovery using relevant prognostic tools (e.g. Neck Disability Index, 12.8%). Over half of the claimants (59.0%) were referred to other professionals at 9–12 weeks post-injury, among which 31.2% were to psychologists and 68.8% to specialists (surgical specialists, 43.6%; WAD specialists, 20.5%). Legal representation and lodgment of full claim were associated with increased number of medical visits and imaging (ρ 0.23 to 0.3; *p* < 0.01).

**Conclusion:**

There is evidence of positive uptake of some guideline recommendations by insurers and health professionals; however, there are practices that are not compliant and might lead to poor health outcomes and greater treatment cost. Organisational, regulatory and professional implementation strategies may be considered to change practice, improve scheme performance and ultimately improve outcomes for people with WAD.

**Electronic supplementary material:**

The online version of this article (10.1186/s12913-018-3439-5) contains supplementary material, which is available to authorized users.

## Background

In primary musculoskeletal health care, many elements of practice have been identified as being ineffective and costly. Such practices include overuse of imaging and overuse of passive interventions [1–5]. Accordingly, clinical practice guidelines have been developed and implemented to promote an evidence-based approach to patient care. These guidelines advocate a biopsychosocial approach with emphasis on secondary prevention of disability and promotion of self-management [6]. Recommendations therefore include; diagnosis based on triage, limited and targeted use of imaging, provision of active (performed by the patient requiring volitional effort) rather than passive (performed by a therapist with no patient effort) treatments, defined timeframes for review for an episode of care and appropriate referral of people who are not recovering. However, guideline implementation and changing professional practice remain a challenge in health care [7–9]. Prior research has shown that implementation strategies had variable effects in changing practice and where effective, these strategies appeared to influence only a few elements of practice [10–15]. Further, effectiveness of these strategies has been evaluated between 3 to 12 months after the intervention and long-term practice change has not been assessed. This is important because sustaining change in practice ensures quality care through continued provision of best treatments and efficient use of allocated resources [16–18].

Implementation strategies for musculoskeletal guidelines have shown positive effects on practices such as referral for imaging and provision of active treatments. Requests for x-rays have reduced with strategies such as distribution of educational materials, educational meetings and reminder messages [19–24]. .Health professionals more frequently advised active treatments in the management of neck pain and low back pain after delivery of a multi-faceted strategy involving educational meetings [13]. Further, the number and duration of physiotherapy visits decreased after a continuing education course and subsequent educational meetings on evidence-based management for neck pain were implemented [25]. Similarly, in arthritis management, improvements in provision of education, joint protection, social support, weight management, onward referral and prescription of medication have been observed [26].

In New South Wales (NSW) Australia, clinical practice guidelines for whiplash associated disorders (WAD) have been developed, updated and implemented over the past 17 years by the state insurance regulator, State Insurance Regulatory Authority (SIRA) [27–29]. WAD is a huge health and economic burden in Australia with 50% of people with WAD experiencing persisting pain and disability [30]. Annual costs for WAD are more than $950 million [31] and in NSW, 46% of all claims lodged since 2007 were attributed predominantly to WAD [29]. The WAD guidelines assist health professionals to deliver best care and insurers in their decision about funding best care [27–29]. The key guideline messages about assessment, interventions, prognosis, review and referral have been consistent through all iterations of the guidelines [27–29]. The WAD guidelines have been disseminated since their inception with an extensive and strategic implementation program. This program has included dissemination through professional associations [32, 33], educational workshops in insurance companies [34], education by opinion leaders [35, 36] and online education [37].

Consistent with results of studies in other musculoskeletal conditions, implementation strategies for WAD guidelines to date have resulted to varying success in changing professional practice of insurers and health professionals. Awareness of and compliance with the guidelines have increased among insurance staff and health professionals have improved provision of active treatments, reassurance and classification of WAD following an educational session led by opinion leaders [34–37]. However, there was no change demonstrated on use of appropriate outcome measures and identification of poor prognosis [36]. Compliance with recommendations on use of imaging, passive interventions and providing associated clinical care pathways have also not been explored. These elements of practice have therefore been targeted in the implementation of the most recent version of the WAD clinical practice guidelines released in 2014 [29].

Full implementation of guidelines typically takes 3 years [9] and the WAD guidelines have been actively implemented since 1999. It is therefore timely to ascertain whether insurers and health professionals continue to provide guideline-based care to people with WAD. One method to evaluate compliance with guidelines is through an audit of practice. Audit of practice has been utilised in previous studies to measure professional practice change [21, 35, 36, 38–40], with this method argued as one of the more robust methods to evaluate quality of the process care [41, 42]. Process of care data involve aspects of the health professional-patient encounter and can be obtained from various sources including clinical notes, patient forms and insurer records [43]. Audit of insurer approvals and disapprovals and the timing of these decisions can also provide insight into indicators of scheme performance.

In NSW Australia, submission of claim-related documentation such as claim forms (e.g. accident notification form (ANF), personal injury claim form (PICF)) and physical treatment request forms (notification of commencement (NOC) form, review form) are the primary mechanisms for communicating details of management to insurers and accessing treatments after WAD. A systematic evaluation of claim-related documentation provides a picture of the extent to which insurers and health professionals are implementing the WAD guidelines. An audit of claim-related documentation will also allow for exploration of other factors that could influence change in clinical practice. Factors such as legal representation, compensation and claim type have been associated with increased health care utilisation following a motor vehicle crash (MVC) [44–46]. Further, comparing cross-sectional audits of practice conducted in similar cohorts across different time points will also help determine which changes in practice have been sustained over time.

This study therefore aimed to determine compliance of insurers and health professionals with the recommendations of the 2014 clinical practice guidelines for WAD and examine whether changes in professional practice have been sustained over time in light of previous investigations of WAD guideline implementation. In addition, this study aimed to explore claim-related factors associated with guideline compliance. Knowledge of compliance with guidelines in practice will form the basis for improving injury management processes and informing future implementation strategies.

## Methods

### Study design

This was an observational study that involved an audit of claimant files. Ethics approval was given by the Northern Sydney Local Health District Human Research Ethics Committee (LNR/16/HAWKE/78).

### Setting

Four compulsory third party insurers in NSW, Australia agreed to participate in this study. New South Wales operates under a common law fault-based scheme where injury management costs are covered by compulsory third party (CTP) insurance. CTP insurance is underwritten by private insurance companies regulated by a government authority (SIRA). To be eligible for benefits people with WAD can submit a claim either within 28 days (ANF) or within 6 months (PICF) of the accident [47]. ANF allows early without prejudice access to treatments and payment for lost earnings up to $5000 through acceptance of provisional liability. PICF covers expenses more than $5000 and applies to those whose recovery would take longer than six months. Once liability is determined, requests for treatments are approved or declined on the basis of whether requests are reasonable and necessary. This can be determined through consideration of factors such as relationship of the service to the accident, benefit to the claimant, appropriateness of the service, appropriateness of the provider and cost [48]. Accordingly, there are no restrictions on the number and type of treatments permitted.

### Sample

A random sample of 288 files, selected based on the relative market share of the insurers, was deemed to be representative. The sample was calculated from 1146 new claims submitted between 1 August 2015 and 15 November 2015, more than 6 months from the release of the most recent version of the guidelines in December 2014. This was necessary to provide adequate time for implementation and uptake of the recommendations. Inclusion criteria were; (1) whiplash as primary injury, (2) age of claimant > 17, and (3) no fatality reported.

### Data source

Primary sources of data were claim forms (e.g. ANF, PICF), medical certificate, NOC and review forms, and insurer file notes. The ANF and PICF, accompanied by a medical certificate, outline demographic and injury details, results of general medical practitioner (GP) assessment, request for imaging, referral to physical treatment (e.g. physiotherapy, chiropractic, etc.) and medications [49]. The NOC and review forms detail the physical treatment provider’s treatment plan and requests for imaging and referral to other professionals [49]. Other data sources included practitioner reports, imaging reports, and invoices. Files were identified from the central database used by SIRA for claims management purposes and were made available to the research team in either paper or electronic format.

### Data collection

Four researchers attended the participating insurer offices to extract data using a standard form. The standard form was a password-encrypted Excel spreadsheet created by the researchers to obtain relevant information to address the aims of the study. Another researcher randomly extracted data from 20% of all files to compare for agreement with the data extracted by other researchers. Fortnightly meetings were held to resolve disagreements and ensure consistency of data extraction.

Data extracted included demographic, claim and injury details, use of health services, and health professional and insurer practices. Demographic information included age, gender, employment status and postcode. Claim details included date of claim lodgement, claim status, claim type (i.e. ANF, full claim direct, full claim converted from ANF) and legal representation. Injury details included accident date and road user type. Use of health services provided information about access to recommended treatments and performance of the scheme and included timing and numbers of medical and physical treatments. Insurer and health professional practice related to compliance with guideline recommendations on classification, use of radiology, prognosis, treatment and referral (Additional file 1: Appendix 1) [29].

#### Insurer compliance

Insurer compliance with guideline recommendations was determined by extracting data on; requests made to the health professional for a Quebec Task Force (QTF) WAD grade (Additional file 1: Appendix 2), insurer-initiated screening of prognostic factors and referral to other health professionals. Information in relation to approval and denial of treatment (Table 4), imaging and referral requests was extracted. The time for insurer response to health professionals’ treatment, imaging and referral requests, and reasons given were also obtained.

#### Health professional compliance

Health professional compliance with guideline recommendations on classification and radiology was determined by extracting data on; diagnostic labels used, and the timing and reasons given for radiological investigations. Compliance with prognosis recommendations was determined by evidence of use of recommended tools (Visual Analogue Scale (VAS), Numeric Rating Scale (NRS)), Neck Disability Index (NDI) [50], Impact of Events Scale (IES) [51] and other). Specific interventions (Table 4) provided were extracted and categorised as; first-line (recommended), adjunctive (not routinely recommended or no evidence), combined first-line and adjunctive, or not recommended. Lastly, data on the timing, reasons given for and outcome of referral to specialist WAD practitioners and/or psychologists were obtained. Specialist WAD practitioners are health professionals with expertise in the management of complex WAD and may include specialist physiotherapists and chiropractors, musculoskeletal medicine practitioners, rehabilitation physicians, pain medicine specialists or occupational physicians [29].

### Data analysis

Data were analysed using descriptive statistics (mean ± SD; median(IQR); frequencies (n(%))). Data for treatment were analysed across the time points; commencement of physical treatment, 6, 12 and 26 weeks post-injury, pertaining to acute, sub-acute and chronic periods of management, respectively. The time points were used as basis to determine whether the number of treatment sessions and provision of adjunctive treatments were tapering over time, for claims with multiple reviews. The researchers made judgements about WAD grade, indications for imaging and appropriateness of referral based on available file information.

Association of demographic factors (WAD grade, employment status) and claim characteristics (claim status, claim type, legal representation) with measures of guideline compliance (number of specialised imaging requests, number GP consults, cost of physical treatment) was determined using correlation analysis. The Shapiro-Wilk statistic revealed that the continuous data were not normally distributed; hence, non-parametric tests of association were used. Correlation coefficients were interpreted as: 0.00 to 0.29, small; 0.30 to 0.49, medium; and 0.50 to 1.0, strong correlation [52]. Statistical analyses were performed using IBM SPSS Statistics v24. Common categories were generated for data entered in text.

## Results

Data extraction occurred from March until October 2016 (Additional file 1: Appendix 3). Demographic, claim and injury details are summarised in Table 1.

**Table 1:** Demographic, claim, injury characteristics from a sample of WAD claims from four NSW CTP insurers

All files	*N* = 288
Demographic characteristics	
Age, mean ± SD	41.6 ± 15.5
Female, n (%)	171 (59.4)
Employment status, n (%)	
Employed	166 (57.6)
Unemployed	90 (31.3)
Self-employed	32 (11.1)
Claim characteristics	
Claim status, n (%)	
Open	263 (91.3)
Finalised	25 (8.7)
Type of claim, n (%)	
Accident notification form	125 (43.4)
Full claim direct^a^	125 (43.4)
Full claim converted^b^	38 (13.2)
Legally represented, n (%)	166 (57.6)
Injury details	
Road user type, n (%)	
Driver	223 (77.4)
Passenger	65 (22.6)
Injury count, median (IQR)	2 (2.0)
WAD grade, n (%)	
Grade I	40 (13.9)
Grade II	230 (79.9)
Grade III	15 (5.2)
Cannot determine	3 (1.0)

*WAD* whiplash associated disorder, *SD* standard deviation, *IQR* interquartile range

^a^ Full claim direct are claims lodged to access benefits more than $5000 and where recovery is expected to last longer than 6 months. A PICF is submitted to the insurance company to access these benefits

^b^ Full claim converted are claims that have initially been submitted through ANF but later on converted to full claim to access further benefits more than $5000

### Classification of whiplash

Insurers requested WAD grade in 6.9% of the files (*n* = 20/288). Health professionals provided WAD grade in 13.0% of the files (*n* = 35/288). The most frequently reported diagnostic label was ‘whiplash injury’ (*n* = 156/288; 54.2%). Use of patho-anatomical terms was observed in 26.7% of the files (*n* = 77/288).

### Request for imaging

Claimants received at least one form of imaging in 57.9% of the files (*n* = 167/288) (Table 2).

**Table 2:** Summary of imaging requests from a sample of WAD claims from four NSW CTP insurers

Imaging requests	Cervical spine x-ray	Specialised imaging
Received imaging, n(%)	*N* = 288	*N* = 288
Done at the emergency department	19 (6.6)	9 (3.1)
Requested by the health professional	62 (21.5)	132 (45.8)
Imaging requested by, n(%)^a^	*N* = 62	*N* = 132
General practitioner	56 (90.3)	100 (75.8)
Allied health practitioner	2 (3.2)	1 (0.8)
Medical specialist	1 (1.6)	11 (8.3)
Other specialist	2 (3.2)	0 (0)
More than one health professional	0 (0)	16 (12.1)
No record	1 (1.6)	4 (3.0)
Health professional justification, n(%)^a^	*N* = 62	*N* = 132
Neck pain, tenderness, loss of motion	22 (35.5)	34 (25.8)
Motor vehicle accident	11 (17.7)	9 (6.8)
Exclude fracture	4 (6.5)	0 (0)
Radiculopathy, numbness, paraesthesia	2 (3.2)	28 (21.2)
Headache, dizziness, nausea	1 (1.6)	5 (3.8)
History of other conditions, prior injury	1 (1.6)	3 (2.2)
Whiplash	1 (1.6)	5 (3.8)
As advised by specialist	0 (0)	5 (3.8)
None cited	20 (32.3)	43 (32.6)
Timeframe (days), median (IQR)^a^	*N* = 62	*N* = 132
Time to request	6.5 (24)	45 (123)
Time to receipt	25 (34)	54.5 (102)

*WAD* whiplash associated disorder, *IQR* interquartile range

^a^ from claimants receiving imaging outside of emergency department

#### Plain radiograph (cervical spine x-ray)

The median time to insurer response to x-ray requests was 22 days. Five files (8.1%) had documented approval of request to rule out cervical fracture and 5 requests (8.1%) were declined for lack of clinical indications. There was no record of insurer response to the request in 79.0% (*n* = 49/62) of the files.

X-rays were conducted for 28.1% of the claimants (*n* = 81/288). For x-rays conducted outside of emergency department (ED), 90.3% of the requests were made by the GP (*n* = 56/62) due to neck pain, tenderness, loss of motion (*n* = 22/62; 35.5%).

#### Specialised imaging

The median time to insurer response to specialised imaging requests was 15 days. Approval was given for 35.6% of the requests (*n* = 47/132) to clarify diagnosis. Twenty-one (15.9%) requests were declined for lack of clinical indications. There was no record of insurer response to the request in 34.1% of the files (*n* = 45/132).

**Fig. 1:**
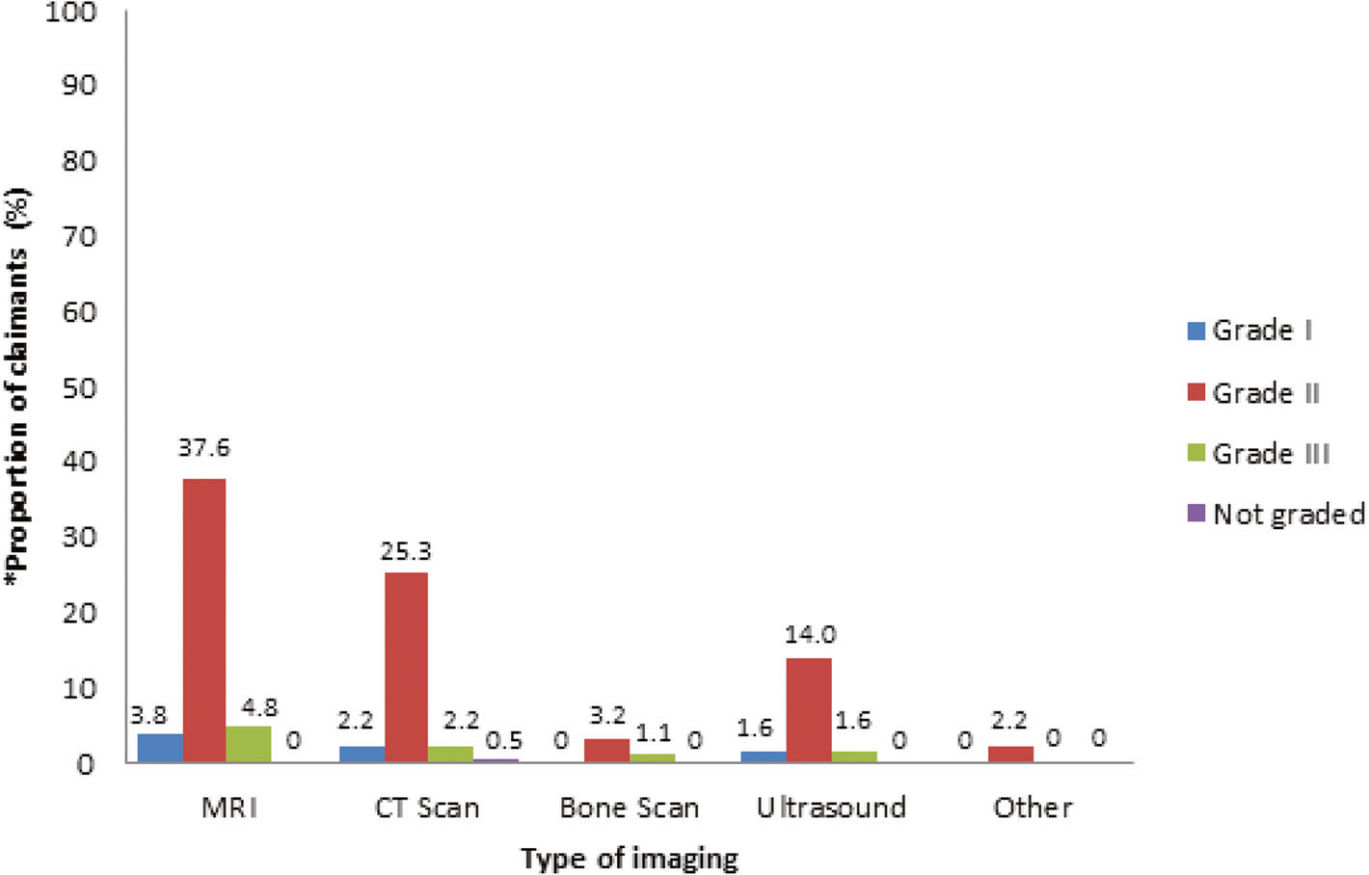
Type of specialised imaging received by WAD claimants according to WAD grade. WAD, whiplash associated disorder; MRI, magnetic resonance imaging; CT, computed tomography. * data obtained from all records of specialised imaging received by claimants (*N* = 186)

Almost half of the claimants received specialised imaging (*n* = 141/288; 48.9%), with 80 claimants receiving at least one specialised imaging and 61 receiving more than one specialised imaging. The majority of specialised imaging was done outside of ED (*n* = 132/141; 93.6%) and requested by GPs (*n* = 100/132; 75.8%) for reasons such as neck pain, tenderness, loss of motion (*n* = 34/132; 25.8%). Magnetic resonance imaging (MRI) was most commonly requested (*n* = 86/186; 46.2%). The majority of those that received specialised imaging were claimants classified as WAD Grade II (Fig. 1).

Based on the review of all files and researcher judgment, 31.8% (*n* = 42/132) of specialist imaging requests were considered compliant with the guidelines given presence of symptoms requiring further imaging to rule out possible neurological pathology.

### Assessment of prognostic indicators

Insurers assessed prognostic indicators using recommended tools in less than 10% of the files (e.g. NRS; *n* = 18/288; 6.3%) (Table 3). Similarly, health professionals assessed these in 1.4% (e.g. IES; *n* = 4/288) to 36.5% (e.g. VAS or NRS; *n* = 105/288) of the files (Table 3). Health professionals also nominated other tools (e.g. Orebro Musculoskeletal Pain Questionnaire [53]) and factors (e.g. complex symptoms) to identify risk of non-recovery in 11.1 and 34.4% of the files, respectively.

**Table 3:** Assessment of prognostic indicators from a sample of WAD claims from four NSW CTP insurers

	Insurer	Health professional
	*N* = 288	*N* = 288
Pain (VAS/NRS), n(%)	18 (6.3)	105 (36.5)
Disability (NDI) [50], n(%)	0 (0)	37 (12.8)
Expectation of recovery, n(%)	24 (8.3)	7 (2.4)
Impact of events (IES) [51], n(%)	0 (0)	4 (1.4)
Other prognostic tools, n(%)		32 (11.1)
Orebro Musculoskeletal Pain Questionnaire [53]		6 (2.1)
Patient Specific Functional Scale [89]		4 (1.4)
Depression, Anxiety, Stress Scales 21 [90]		6 (2.1)
Copenhagen Neck Functional Disability Scale [91]		2 (0.7)
Whiplash Disability Questionnaire [92]		2 (0.7)
Oswestry Low Back Pain Questionnaire [93]		3 (1.0)
Roland Morris Disability Questionnaire [93]		5 (1.7)
Functional Rating Index [94]		4 (1.4)
Other factors identified, n (%)		99 (34.4)
Complex symptoms		54 (18.8)
Multiple injuries		14 (4.9)
Age		3 (1.0)
Nature of work		9 (3.1)
Previous neck injury		5 (1.7)
Delayed treatment		5 (1.7)
Poor fitness/health prior to injury		3 (1.0)
Chronicity of the condition		4 (1.4)
Medication		1 (0.3)
High impact collision		1 (0.3)

*VAS* Visual Analogue Scale, *NRS* Numeric Rating Scale, *NDI* Neck Disability Index, *IES* Impact of Events Scale

### Treatments provided

#### Access to health care services

**Fig. 2:**
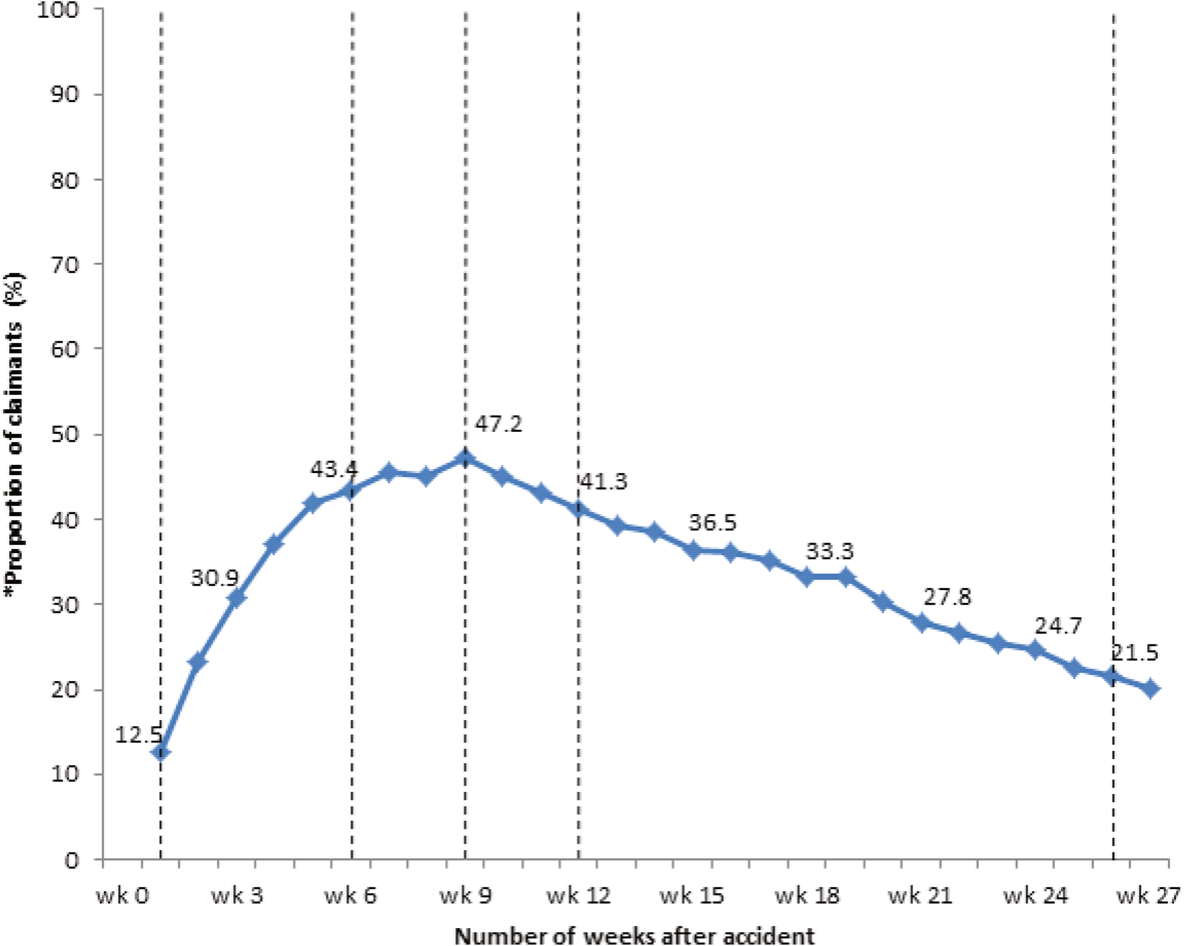
Proportion of WAD claimants accessing physical treatment from the time of accident. * data obtained from all files (*N* = 288)

The median time to GP consult was 4 days after injury and 25 days for physical treatment. The average number of GP consults was 5 sessions and 15 sessions for physical treatment. The majority of claimants (*n* = 250/288; 86.8%) were referred for physical treatment, of which 237 (94.8%) had documented evidence of receiving treatment. The most common health professionals referred to were physiotherapists (*n* = 187/237; 78.9%). Approximately 13% (*n* = 36/288) of claimants received physical treatment 1 week post-injury. The highest proportion of claimants receiving physical treatment was at 9 weeks post-injury (*n* = 136/288; 47.2%) (Fig. 2), which decreased to 21.5% (*n* = 62/288) at 26 weeks.

The median time to insurer response to physical treatment requests was 20 days after receipt of the NOC forms. Insurers approved 93% (*n* = 187/201) of physical treatment requests to promote early treatment, self-management and progress to functional recovery. Physical treatment request was denied in 5% (*n* = 9/201) of the files for reasons including lack of evidence for physical benefit.

#### Guideline-based treatment

**Fig. 3:**
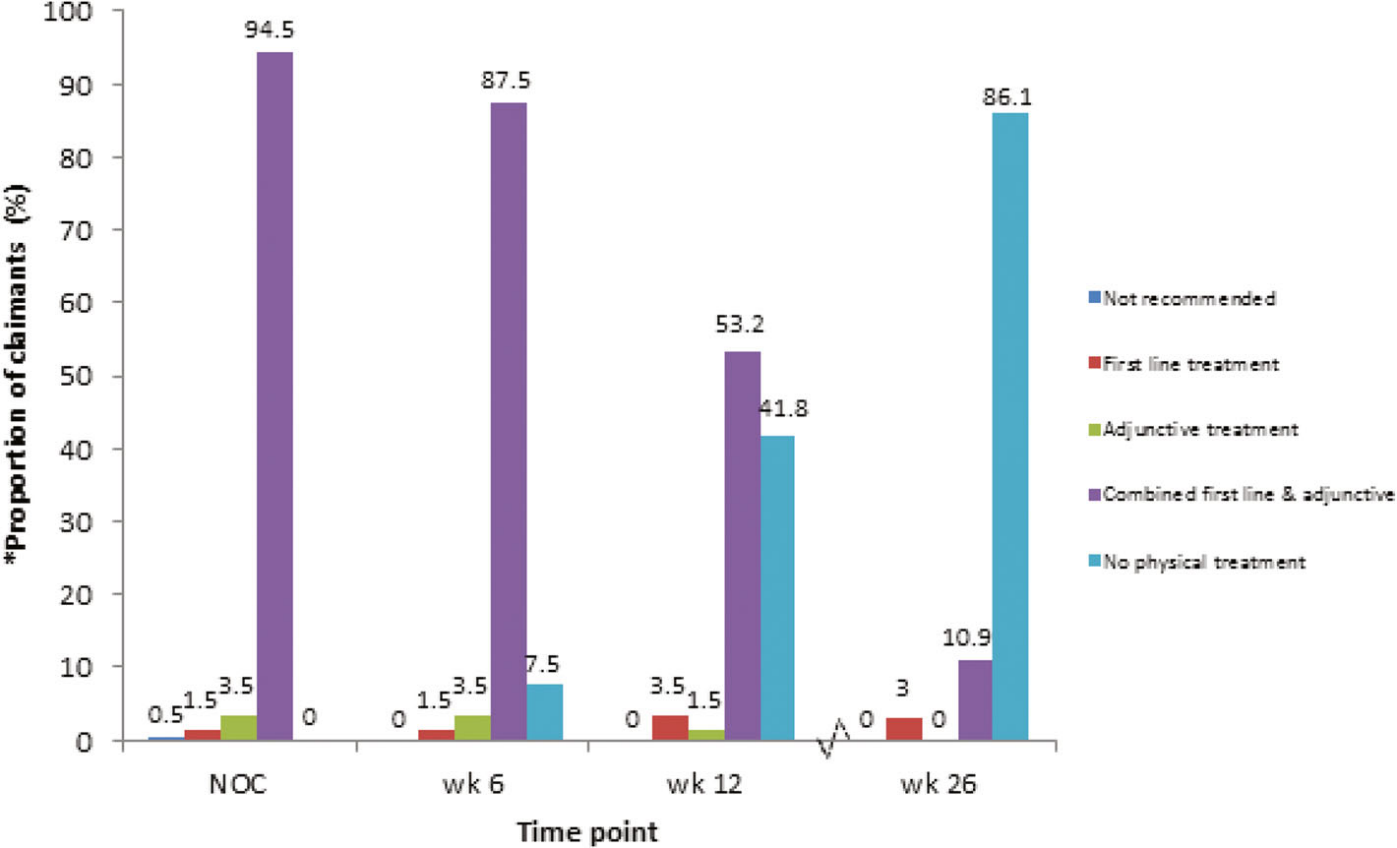
Type of treatments received by WAD claimants over a period of 6 months. * data obtained from claimants receiving physical treatment with documentation submitted (*N* = 201)

Few people with WAD received first-line treatments only (*n* = 3/201; 1.5%) and the majority received a combination of first-line and adjunctive treatments (*n* = 190/201; 94.5%) (Fig. 3). This finding was consistent for claimants who were still undergoing treatments at 6, 12, and 26 weeks after commencement of physical treatment. The majority of claimants received neck exercises (*n* = 188/209; 90%), advice (*n* = 165/205; 80.5%) and manual therapy (*n* = 189/201; 94%) (Table 4). Few received treatments that are not recommended.

**Table 4:** Guideline-based interventions from a sample of WAD claims from four NSW CTP insurers

			Physical treatment	All files
	Grade^a^	N	% *N*=201	% *N*=288
Recommended- evidence of benefit				
Neck Exercises	B	188	90.0^ɸ^	65.3
Advice	B	165	80.5^^^	57.3
Reassurance	B	50	24.5^δ^	17.4
Pain-relieving medications				
Non-steroidal anti-inflammatory drugs	☑	106		36.8
Simple analgesics	☑	95		33.0
Opioids	☑	64		22.2
Not routinely recommended- limited evidence				
Manual therapy	C	189	94.0	65.6
Trigger point needling	D	19	9.5	6.6
Acupuncture	D	14	7.0	4.9
Taping	C	14	7.0	4.9
No evidence of benefit or harm				
Electrotherapy	☑	71	35.3	24.7
Massage	☑	33	16.4	11.5
Heat/Ice	☑	17	8.5	5.9
Traction	☑	3	1.5	1.0
Pilates/Yoga	☑	3	1.5	1.0
Pillow	☑	2	1.0	0.7
Cupping	☑	1	0.5	0.3
Not recommended-evidence of no benefit				
Anti-depressant	☑	14		4.9
Intra-articular and intrathecal steroid injection	☑	10		3.5
Muscle relaxant	B	8		2.8
Collar	A	8		2.8
Anti-convulsant	☑	7		2.4
Reduction of usual activities for more than 4 days	☑	0		0
Botulinum toxin type A	A	0		0
Pulsed electromagnetic treatment	☑	0		0

*WAD* whiplash associated disorder, *NSW* New South Wales, *CTP* compulsory third party, *IQR* interquartile range

^ɸ^*N* = 209; ^^^*N* = 205; ^δ^*N* = 204

^a^Grade of recommendation [29] = A, body of evidence can be trusted to guide practice; B, body of evidence can be trusted to guide practice in most situations; C, body of evidence provides some support for recommendations but care should be taken in its application; D, body of evidence is weak and recommendation must be applied with caution; ☑, consensus recommendation supported by all members of the working group as a graded recommendation could not be made due to lack of evidence

#### Treatment frequency

**Fig. 4:**
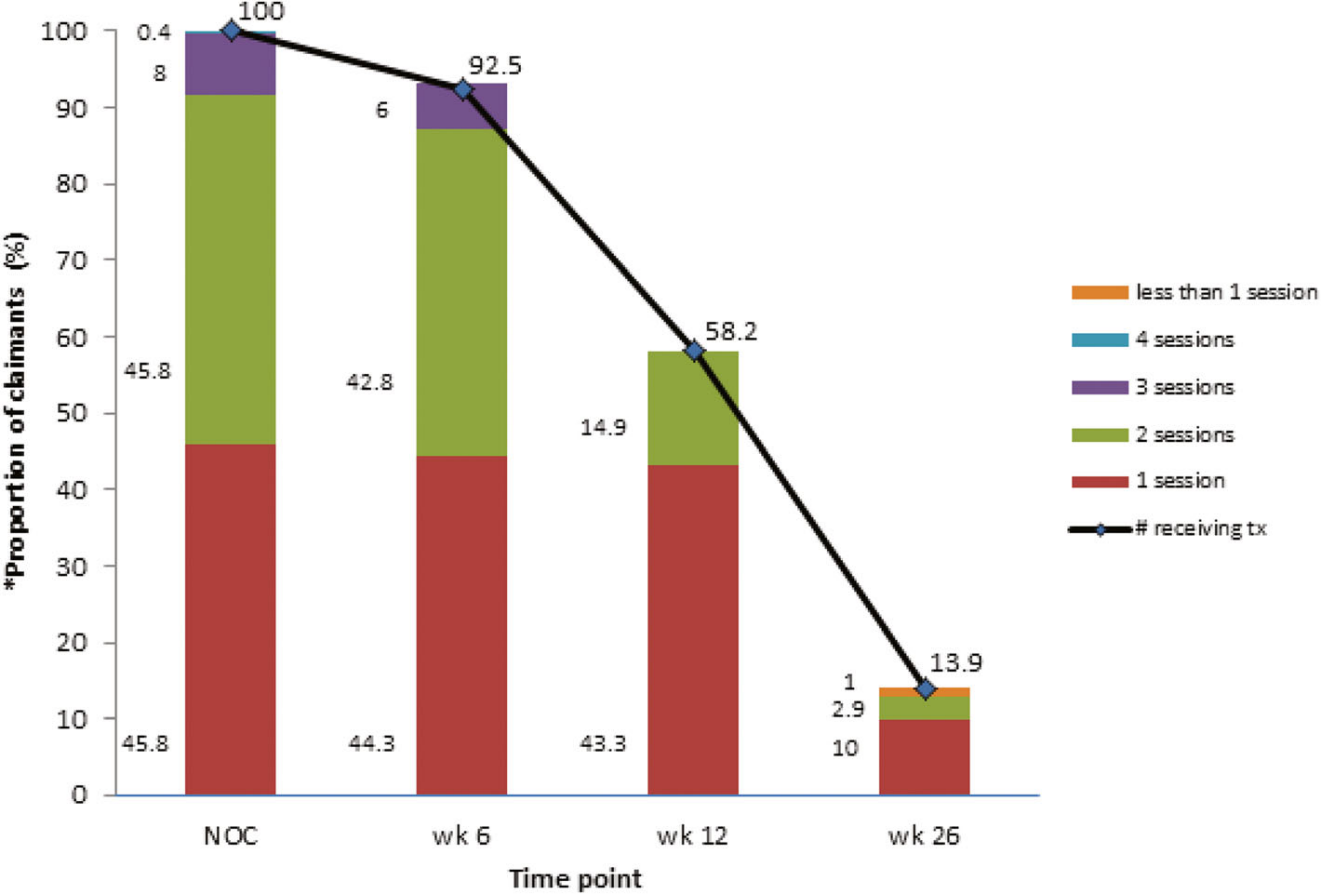
Proportion of WAD claimants receiving treatment and treatment frequency over a period of 6 months. * data obtained from claimants receiving physical treatment with documentation submitted (*N* = 201)

At the commencement of physical treatment, 45.8% (*n* = 92/201) of claimants had one session of physical treatment per week and 45.8% (*n* = 92/201) had 2 sessions per week (Fig. 4). There was evidence of tapering of treatment frequency over time, with the greatest reduction observed at 12 weeks after commencement of physical treatment. At 26 weeks, ~ 10% of claimants were still having physical treatments once a week.

### Referral to other professionals

**Fig. 5:**
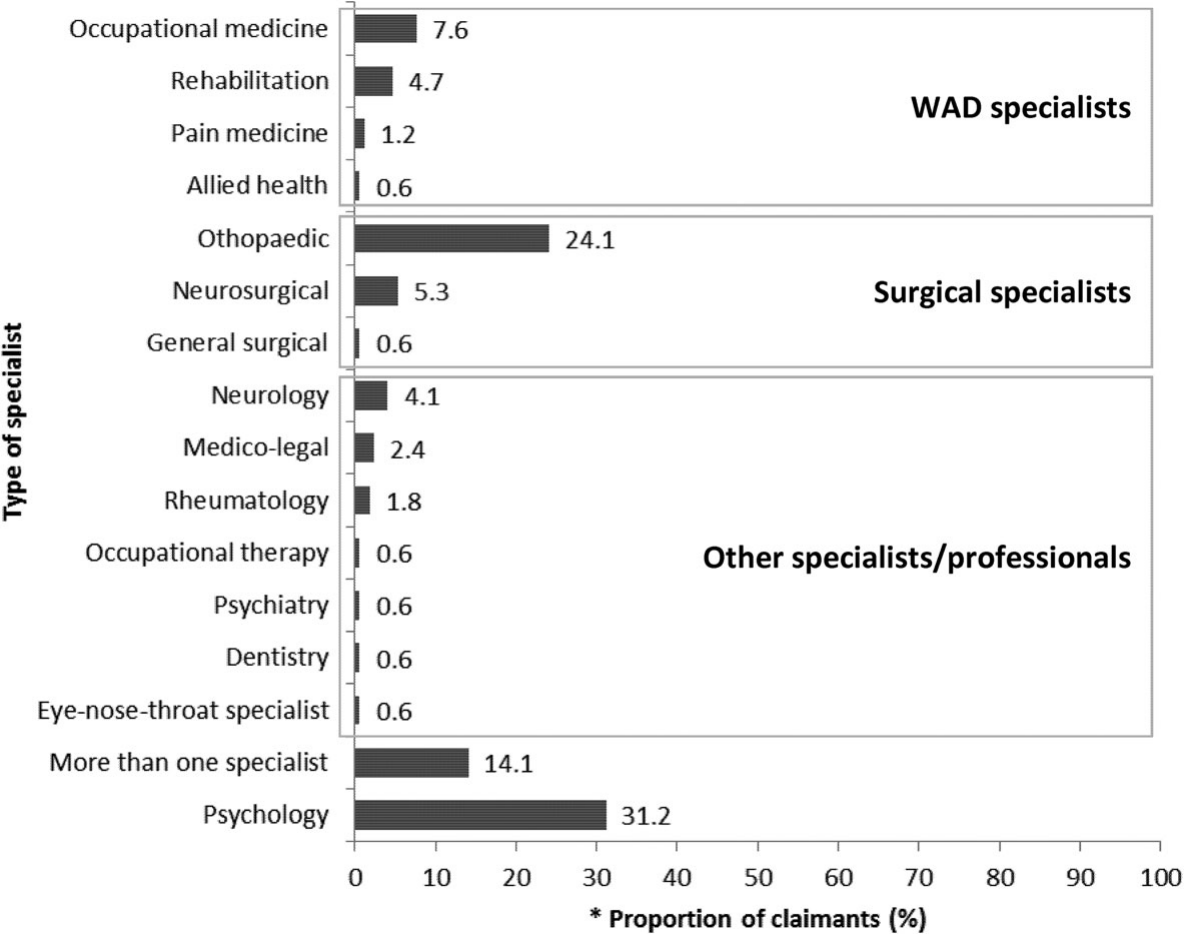
Referral to other professionals from a sample of WAD claims from four NSW CTP insurers. * data obtained from claimants who were referred to other professionals (*N* = 170)

Over half of the claimants (*n* = 170/288; 59.0%) were referred to other professionals (Fig. 5).

#### Specialist referral

The median time to insurer response to specialist referral was 18 days. Request for specialist referral was approved in 20.5% of the files (*n* = 24/117) to confirm diagnosis, assess current status, review appropriateness of treatment and provide recommendations on rehabilitation needs. Insurers declined 10% of specialist referrals (*n* = 11/117) due to lack of indicators for specialist consult.

One-hundred-seventeen claimants (*n* = 117/170; 68.8%) were referred to specialists (Table 5). Of these, 43.6% (*n* = 51/117) involved referral to surgical specialists and 20.5% (*n* = 24/117) to WAD specialists. The main justification for specialist referral was to obtain opinions about diagnosis and management (*n* = 63/117; 53.8%). The median time for referral to specialists was 89 days. Outcomes of referral included clarification of condition (*n* = 26/117; 22.2%) and request for further imaging and tests (*n* = 11/117; 9.4%).

**Table 5:** Referral to specialists from a sample of WAD claims from four NSW CTP insurers

	N	Referred %	All files %
		*N* = 117	*N* = 288
Type of specialist			
Surgical specialists	51	43.6	17.7
WAD specialists	24	20.5	8.3
Other specialists	18	15.4	6.3
More than one specialist	24	20.5	8.3
Referral source			
General practitioner	62	53.0	21.5
Insurer	32	27.4	11.1
Solicitor	16	13.7	5.6
Medical specialist	5	4.3	1.7
Physiotherapist	2	1.7	0.7
Health professional justification			
Opinion about diagnosis and management	63	32.5	21.9
Complexity and severity of the injury	8	6.8	2.8
Persistent pain, non-recovery	6	5.1	2.1
Surgical management, specialised procedures	2	1.7	0.7
Needs assessment	2	1.7	0.7
None cited	36	30.8	12.5
Indicated for WAD specialist but no referral made	55		19.1
Timeframe (days), median(IQR)			
Time to request	89(158)		
Outcome of specialist referral			
Clarification of condition, assessment of impairment	26	22.2	9.0
Further imaging and tests	11	9.4	3.8
Continuation of current management	8	6.8	2.8
Pain management (medications, injections)	6	5.1	2.1
Referral to another health professional	5	4.3	1.7
Rehabilitation plan	2	1.7	0.7
Discontinuation of treatment	2	1.7	0.7
Return to work	2	1.7	0.7
No record	55	47.0	19.1

*WAD* whiplash associated disorder, *NSW* New South Wales, *CTP* compulsory third party, *IQR* interquartile range

Based on review of files and researcher judgment, an additional 19.1% of claimants (*n* = 55/288) would have benefited from WAD specialist consult where no referral was made. These indications were obtained from reports of apparent non-recovery at review and presence of high-risk factors, such as high pain and disability.

#### Psychologist referral

The median time to insurer response to psychology referral was 44 days. Insurers approved 54.7% of the referral requests (*n* = 29/53) to address psychological needs and declined 13.2% of the requests (*n* = 7/53) due to lack of indicators.

Fifty-three claimants (*n* = 53/170; 31.2%) were referred to psychologists due to presence of anxiety and fear of driving (*n* = 22/53; 41.5%) (Table 6). The median time for referral to psychologists was 63 days.

**Table 6:** Referral to psychologists from a sample of WAD claims from four NSW CTP insurers

	N	Referred % *N* = 53	All files % *N* = 288
Referral source			
General practitioner	42	79.2	14.6
Medical specialist	6	11.3	2.1
Insurer	3	5.7	1.0
Physiotherapist	1	1.9	0.3
Solicitor	1	1.9	0.3
Justification for psychologist referral			
Fear of driving, anxiety	22	41.5	7.6
Post-traumatic stress disorder	7	13.2	2.4
History of psychological problem	4	7.5	1.4
Depression	3	5.7	1.0
Non-recovery	3	5.7	1.0
Flashbacks, insomnia	2	3.8	0.7
Shock, distress	2	3.8	0.7
None cited	10	18.9	3.5
Indicated for psychology but no referral made	51		17.7
Timeframe (days), median (IQR)			
Time to request	63(76)		

*WAD* whiplash associated disorder, *NSW* New South Wales, *CTP* compulsory third party, *IQR* interquartile range

Based on review of files and researcher judgment, 73.6% (*n* = 39/53) of the referrals made to psychologists were considered appropriate to address psychological symptoms. There was also a proportion of claimants deemed that would have benefited from psychologist consult due to reported presence of psychological symptoms (*n* = 51/288; 17.7%); however, no referral was made.

### Association of claim factors with measures of guideline compliance

There was moderate correlation between full claim and increased number of specialised imaging received (*ρ* = 0.30, *p* < 0.01), and between open claim and increased number of GP consults (*ρ* = 0.30, *p* < 0.01). A small correlation was observed between legal representation and increased number of specialised imaging received (*ρ* = 0.24, *p* < 0.01) and GP consults (*ρ* = 0.23, *p* < 0.01), and higher total cost of physical treatment (*ρ* = 0.23, *p* < 0.01).

## Discussion

This study shows that health professionals have adopted some of the guideline recommendations when assessing and treating people with WAD. However, there are some aspects of insurer and health professional practice that are not aligned with key recommendations of the guidelines. Specialised imaging and passive treatments are being provided and approved in a large proportion of claims and monitoring of prognosis and appropriate specialist referral are not being sought by providers or insurers in a large proportion of claims. These practices have been identified as priority areas for improvement in light of the new legislation, the Motor Accidents Injuries Act 2017, which limits benefits for minor injuries such as WAD to 6 months. In the following discussion, suggestions will be made regarding strategies to facilitate compliance with recommendations of the WAD guidelines to ultimately improve outcomes for WAD.

These findings suggest that previous implementation strategies and guideline review since 1999 have been effective in changing aspects of WAD management [10, 34–37]. Rates of x-ray investigation in this study (21.5%) were relatively low compared to the rates reported from the year 2000 to 2009 (30.0%) [54]. This is consistent with previous work in Canada and Australia where rates of x-ray investigations following WAD reduced following education and training of physicians, ED staff and nurses on use of the Canadian C-spine rule [55]. In addition, neck exercises and advice to keep active are being provided by health professionals and approved by insurers for the majority of claimants, demonstrating widespread acceptability of these guideline recommendations. Positive results of implementation strategies support the value of continuing education among insurers and health professionals about WAD guidelines.

These results also reveal that insurer claims management processes can facilitate early access to and approval of treatments and contribute to improved scheme performance. Claimants are using the ANF type of claim effectively, with 56% of claims lodged initially through this system and approved by insurers. Moreover, the median time to commencing physical treatment was 3 weeks post-MVC. These results show that claimants are taking advantage of provisional liability and accessing treatments earlier than they would have if they had to wait for full admission of liability. In a study comparing outcomes and costs for WAD pre and post the 1999 legislative reform in NSW, medical expenses in the first 6 months post-injury were higher at 2 and 4 years after the reform, indicating early access to treatment was achieved [56]. This study suggests compliance with legislative intent of the Motor Accidents Compensation Act 1999 to provide early access to treatments for people with WAD.

Whilst the current study found evidence of compliance by insurers and health professionals with some guideline recommendations, other observed practices such as not using the QTF WAD classification system and unnecessary specialised imaging demonstrate non-compliance. Health professionals commonly used “whiplash injury” (54.2%) or patho-anatomical diagnoses (26.7%), rather than the QTF classification. Further, high rates of MRI were observed despite majority of claimants being classified as Grade II, which is consistent with pursuing a patho-anatomical diagnosis. Use of unnecessary imaging in WAD has also been observed in Victoria, Australia [54], and in American and Australian cohorts investigating low back pain [57, 58]. This practice is not recommended given the lack of association between imaging findings and clinical symptoms [59–65], and adverse outcomes associated with unnecessary imaging [66–69]. The patterns observed in the current study and that of others suggest a dominant biomedical approach to management, which conflicts with the biopsychosocial approach advocated by the guidelines.

The high proportion of claimants receiving passive treatments beyond the acute phase of injury (> 12 weeks) may also reflect an approach that is inconsistent with guideline recommendations. Manual therapy is often provided for people with WAD in other countries [70, 71], suggesting that a proportion of health professionals continue to utilise a symptom-focused approach in management. Whilst the provision of manual (passive) therapy is recommended as an adjunct to active treatment in the acute phase of WAD, the persistence of providing this without evidence of benefit is not recommended as it promotes passive coping strategies and poor self-efficacy [29, 72–74]. Given both these factors are associated with non-recovery [75, 76], empowering the injured person to manage their symptoms is recommended to improve health outcomes [6].

Further, both insurer and health professional practices demonstrate non-compliance with identification of claimants at risk of non-recovery and acting on this with timely and appropriate referral. Health professionals and insurers inconsistently used validated prognostic tools and rarely reported on expectation of recovery. Most referrals were made to surgical specialists, arguably too late (> 13 weeks) to influence the course of recovery as studies on WAD recovery trajectories demonstrate that most recovery occurs within 12 weeks of injury [30, 77–79]. Additionally, recent legislative reform in NSW (Motor Accidents Injuries Act 2017 No 10) will limit access to benefits to 6 months for people with minor injuries such as WAD. Thus, the recommendation in guidelines is for onward referral between 3 and 6 weeks to address non-recovery [29]. The change in the scheme, guideline recommendations on referral and data on recovery trajectories emphasize the importance of ensuring treatments maximise the potential for recovery and limit chronicity.

Given that implementation strategies to date (e.g. dissemination of guidelines, educational workshops, online education) have not been completely successful in promoting compliance with all of the recommendations in the guidelines, alternative strategies may need to be considered. One strategy may be to implement organisational changes such as mandating practice through policy or within insurer claims management processes. Regulatory or legislative changes may also be implemented by the insurance regulator to involve restrictions in payment of treatment requests or having a reasonable cap for number of treatments provided. Evidence in other settings showed that changing policies at the level of the organisation resulted to changes in practice. Reimbursement restriction policies decreased inappropriate medication prescribing [80], and promoted better use of medications with reduced costs without an increase in utilisation of other health services [81].

At the level of the insurers, changes in claims management processes may include mandated use of validated risk screening tools, such as the clinical prediction rule for WAD [82, 83], and mandated peer review by WAD specialists based on risk of non-recovery. Such practices have demonstrated effect; implementing use of screening tools such as the Canadian C-spine rule as a policy within a community hospital ED led to a significant reduction in x-ray requests [84]. Further, results of a study among injured workers showed that mandated risk assessment and early referral to psychologists of those at high-risk of delayed recovery improved outcomes and reduced costs [85]. A qualitative study exploring the perceptions of health professionals on WAD specialist referral also suggested that a standardised process for specialist review mandated by the insurers might promote good referral practices [86]. Another strategy could be to use WAD-specific standard reporting and referral forms, a strategy shown to improve compliance in other health populations [87, 88].

Professional implementation strategies have been effective in instigating practice change; however, there are entrenched behaviours remaining unchanged and appeared to be influenced by scheme and compensation factors. Results of the current study showed that having legal representation and full, ongoing claims were associated with higher number of medical visits, specialised imaging and cost of treatment. Although associations were weak to moderate, these results highlight that organisational and regulatory changes, in addition to professional implementation strategies, may be necessary to change practice.

### Limitations

Whilst data were extracted from all available sources, this study was limited by the completeness of the files. The majority of the claimant files were open, ongoing claims at the time of data extraction; hence, some data were not available (e.g. insurer correspondence). Another limitation could be that the files were obtained from new claims submitted approximately 8 months after the release of the most recent WAD guidelines. Insufficient time may have elapsed for the guideline recommendations to be adopted in practice. Additionally, only insurer files were audited; hence, details of specific treatments and management decisions made by health professionals may not have been adequately captured. Lastly, results may not be generalised to practices not operating within the same healthcare and compulsory third party insurance systems as management processes and decisions may differ.

## Conclusion

In summary, there is evidence of insurer and health professional practices that are compliant with recommendations from WAD guidelines. Claimants receive early access to and approval of appropriate care. However, there is also evidence of practices that are not compliant and might lead to poor health outcomes and greater treatment costs. Alternate implementation strategies may need to be considered to reduce unnecessary imaging and the persistent provision of passive treatments; promote use of validated prognostic tools; and maximise early referral to WAD specialists and psychologists. It appears that multiple strategies involving organisational, regulatory and professional interventions may be necessary to successfully implement the recommendations of the WAD guidelines to change practice, improve scheme performance and ultimately improve outcomes for people with WAD.

## Additional file

Additional file 1: **Appendix 1.** Insurer and health professional data collected based on recommendations of the guidelines. **Appendix 2.** Quebec Task Force classification of grades of WAD. **Appendix 3.** Flow diagram of claimant files included in the study. (DOCX 37 kb)

## Abbreviations

ANF: Accident notification form
CTP: Compulsory third party
ED: Emergency department
GP: General medical practitioner
IES: Impact of events scale
IQR: Interquartile range
MRI: Magnetic resonance imaging
MVC: Motor vehicle crash
NDI: Neck disability index
NOC: Notification of commencement
NRS: Numeric rating scale
NSW: New South Wales
QTF: Quebec Task Force
SD: Standard deviation
SIRA: State Insurance Regulatory Authority
VAS: Visual analogue scale
WAD: Whiplash associated disorder
