# Supplementary material for: Adoption and use of guidelines for whiplash: an audit of insurer and health professional practice in New South Wales, Australia
Source: BMC Health Serv Res. 2018 Aug 8;18:622. doi: 10.1186/s12913-018-3439-5 (PMC6083615; doi:10.1186/s12913-018-3439-5)
Supplement: Supplementary file 1 — Appendix 1. Insurer and health professional data collected based on recommendations of the guidelines. Appendix 2. Quebec Task Force classification of grades of WAD. Appendix 3. Flow diagram of claimant files included in the study. (DOCX 37 kb) [file 12913_2018_3439_MOESM1_ESM.docx]

**Appendix 1.** Insurer and health professional data collected based on recommendations of the guidelines

| **Guideline recommendation** | **Insurer data collected** | **Health professional data collected** |
| --- | --- | --- |
| Classification of whiplash | WAD classification based on QTF system | Diagnosis given by the health professional   - - - - WAD classification based on QTF system       - Whiplash injury (no QTF grade)       - Other: soft tissue injury, neck (cervical) sprain/strain, myofascial pain |
| Imaging |  |  |
| Plain radiographs | Decision outcome   - - - - Approved       - Declined       - No record available   Decision reason  Number of days from imaging (plain radiograph, specialised imaging) request to date of insurer decision | Source of x-ray request   - - - - Done at emergency department       - General practitioner       - Medical specialist       - Physiotherapist       - Chiropractor       - Other health professional   Number of days from accident to x-ray request  Number of days from accident to x-ray receipt  Justification for x-ray request |
| Specialised imaging |  | Type of specialised imaging technique requested   - - - - Magnetic resonance imaging       - Computed tomography scan       - Ultrasound       - Bone scan       - Electroencephalography/Electromyography       - Other specialised imaging technique   Source of specialised imaging request   - - - - Done at emergency department       - General practitioner       - Medical specialist       - Physiotherapist       - Chiropractor       - Other health professional   Number of days from accident to specialised imaging request  Number of days from accident to specialised imaging receipt  Justification for specialised imaging request |
| Prognosis | Assessment of pain (VAS, NRS)  Assessment of disability  Assessment of expectation of recovery  Other factors identified by the insurer that would impact on recovery | Prognostic tools used   - - - - Pain rating scale (VAS, NRS)       - Neck Disability Index       - Impact of Events Scale       - Other prognostic tools   Assessment of expectation of recovery  Other factors identified by the health professional that would delay recovery |
| Treatment |  |  |
| Medical services |  | Number of days from accident to medical (GP) consultation  Number of medical (GP) visits  Cost of medical (GP) consult  Type of medications prescribed   - Simple analgesics - Non-steroidal anti-inflammatory drugs - Oral opioids - Anti-depressant - Anti-convulsant - Muscle relaxant |
| Physical treatment | Decision outcome   - - - - Approved       - Declined       - Partially approved       - No record available   Decision reason  Number of physical treatment sessions approved  Number of treatment plans approved  Cost of treatment  Number of days from treatment plan request to date of insurer decision | Physical treatment professional   - Physiotherapist - Chiropractor - Osteopath - Exercise physiologist - Other   Number of days from accident to physical treatment consultation  Type of physical treatment provided   - Education - Self-management - Manual therapy - Supervised exercises - Electrotherapy   Number of physical treatment sessions requested  Duration of physical treatment (number of weeks)  Number of physical treatments received  Cost of physical treatment |
| Referral |  |  |
| Specialists | Decision outcome   - - - - Approved       - Declined       - No record available   Decision reason  Number of days from referral (specialist, psychologist) to date of insurer decision | Type of specialists   - - - - WAD specialist:[6] specialist physiotherapists, specialist chiropractors, musculoskeletal medicine practitioners, rehabilitation physicians, pain medicine specialists or occupational physicians       - Surgical specialist: orthopaedic surgeon, neurosurgeon, general surgeon       - Other specialist: neurologist, psychiatrist, rheumatologist   Number of days from accident to specialist referral  Justification for specialist referral  Outcome of specialist referral |
| Psychologists |  | Number of days from accident to psychologist referral  Justification for psychologist referral  Psychologist diagnosis  Treatment recommendation  Number of psychology treatment sessions  Cost of psychology treatment |

WAD, whiplash associated disorder; QTF, Quebec Task Force; VAS, visual analogue scale; NRS, numeric rating scale; GP, general practitioner

**Appendix 2.** Quebec Task Force classification of grades of WAD

| **Grade** | **Classification** |
| --- | --- |
| 0 | No complaint about the neck.  No physical sign(s). |
| I | Complaint of neck pain, stiffness or tenderness only.  No physical sign(s). |
| II | Neck complaint AND musculoskeletal sign(s).  Musculoskeletal signs include decreased range of movement and point tenderness. |
| III | Neck complaint AND neurological sign(s).  Neurological signs include decreased or absent tendon reflexes, weakness and sensory deficits. |
| IV | Neck complaint AND fracture or dislocation. |

WAD, whiplash associated disorder

Appendix 3. Flow diagram of claimant files included in the study

Eligible files identified from insurance records database (n=1146)

Eligible files that underwent randomisation

(n=1052)

Excluded (n=94)

- Age not >17 (n=7)
- WAD Grade 4 (n=42)
- Pedestrian, motorcycle rider, cyclist, pillion (n=45)

Excluded (n=64)

- Records could not be obtained (n=27)
- Insurer denied liability (n=10)
- Non-NSW resident (n=8)
- Incomplete forms submitted (n=19)

Files included for analysis

(n=288)

Files identified for data extraction

(n=352)

WAD, whiplash associated disorder; NSW, New South Wales
